# Transcriptome remodeling of *Pseudomonas putida* KT2440 during mcl-PHAs synthesis: effect of different carbon sources and response to nitrogen stress

**DOI:** 10.1007/s10295-018-2042-4

**Published:** 2018-05-07

**Authors:** Justyna Mozejko-Ciesielska, Tomasz Pokoj, Slawomir Ciesielski

**Affiliations:** 1 Department of Microbiology and Mycology, Faculty of Biology and Biotechnology, University of Warmia and Mazury in Olsztyn, Oczapowskiego 1A, 10-719 Olsztyn, Poland; 2 Department of Environmental Biotechnology, University of Warmia and Mazury in Olsztyn, Olsztyn, Poland

**Keywords:** Biopolymers, Nitrogen starvation, Quantitative PCR, RNAseq analysis, Transcriptomics

## Abstract

Bacterial response to environmental stimuli is essential for survival. In response to fluctuating environmental conditions, the physiological status of bacteria can change due to the actions of transcriptional regulatory machinery. The synthesis and accumulation of polyhydroxyalkanoates (PHAs) are one of the survival strategies in harsh environments. In this study, we used transcriptome analysis of *Pseudomonas putida* KT2440 to gain a genome-wide view of the mechanisms of environmental-friendly biopolymers accumulation under nitrogen-limiting conditions during conversion of metabolically different carbon sources (sodium gluconate and oleic acid). Transcriptomic data revealed that *phaG* expression is associated with medium-chain-length-PHAs’ synthesis not only on sodium gluconate but also on oleic acid, suggesting that PhaG may play a role in this process, as well. Moreover, genes involved in the β-oxidation pathway were induced in the PHAs production phase when sodium gluconate was supplied as the only carbon and energy source. The transition from exponential growth to stationary phase caused a significant expression of genes involved in nitrogen metabolism, energy supply, and transport system. In this study, several molecular mechanisms, which drive mcl-PHAs synthesis, have been investigated. The identified genes may provide valuable information to improve the efficiency of this bioprocess and make it more economically feasible.

**Electronic supplementary material:**

The online version of this article (10.1007/s10295-018-2042-4) contains supplementary material, which is available to authorized users.

## Introduction

Bacterial survival requires the ability to sense and respond to fluctuating environmental conditions. To sustain growth, microorganisms must constantly cope with upcoming challenges, especially alterations in nutrient availability. Cellular stress response to nitrogen starvation is a complex process that not only affects microbial existence but also influences the productivity of some metabolic products such as polyhydroxyalkanoates [18].

Polyhydroxyalkanoates (PHAs) are bacterial storage compounds produced by many microorganisms under growth-limiting conditions, such as a nitrogen, phosphorus, or oxygen starvation, and when an excess of a carbon source is available in the environment. These biopolymers continue to attract increasing industrial interest as a renewable, biodegradable, biocompatible thermoplastics being considered as a replacement for hazardous petrochemical polymers. Several reviews have described their potential applications [2, 6, 7]. The main groups of PHAs are short-chain-length PHAs (scl-PHAs) with five or less carbon atoms, and medium-chain-length PHAs (mcl-PHAs) with six or more carbon atoms. Scl-PHAs synthesis is a common feature of many microorganisms, whereas mcl-PHAs have been observed mainly in fluorescent and non-fluorescent *Pseudomonas* species. Mcl-PHAs could consist of 3-hydroxyhexanoate (3HHx), 3-hydroxyoctanoate (3HO), 3-hydroxydecanoate (3HD), 3-hydroxydodecanoate (3HDD), 3-hydroxytetradecanoate (3HTD), or even higher chain length monomers, and appear to be much more elastomeric and resistant than scl-PHAs [29, 45]. It is well known that the monomeric composition of PHAs varies depending on the producing organism and on the available carbon source [34, 35].

PHA-producing microorganisms are able to link carbon catabolic pathways together with PHA anabolic pathways, which makes this microorganisms specialized cell factories that can produce PHAs from various carbon sources [46]. These potential carbon sources are saccharides, *n*-alkanes, *n*-alkanoic acids, *n*-alcohols, and various gases (e.g., methane and carbon dioxide). Depending on the type of carbon source, bacteria generally use one of two central pathways for (*R*)-3-hydroxyacyl-CoA generation, either β-oxidation or fatty acid de novo synthesis. β-Oxidation is used when fatty acids and alkanoic acids are used as carbon sources. In contrast, the fatty acid de novo synthesis pathway converts structurally non-related carbon sources, such as glucose, glycerol, gluconate, acetate, or ethanol [22]. The fatty acid de novo biosynthesis pathway is linked with PHA biosynthesis by the *phaG* gene. This gene codes for transacylase, which catalyzes the transfer of the (*R*)-3-hydroxydecanoyl moiety from acyl carrier protein to CoA [21].

The PHA biosynthesis gene cluster of *Pseudomonas* species comprises four genes that encode two PHA synthases (*phaC1* and *phaC2*), an intracellular depolymerase (*phaZ*) and a transcriptional regulator (*phaD*) [25]. Another gene cluster, containing the genes encoding the PHA granule-associated proteins *phaF* and *phaI*, believed to be involved in PHAs biosynthesis regulation, is located downstream of the PHAs synthesis gene cluster and is transcribed in the opposite direction [41]. The *phaG* gene mentioned above is not co-localized with other *pha* genes [20]. Although the key genes engaged in the mcl-PHAs biosynthesis are known, knowledge on the regulatory mechanisms at the transcriptomic level during mcl-PHAs synthesis under nitrogen starvation is limited. Advances in next-generation sequencing technologies provide new opportunities for finding information on microbial physiology and metabolic pathways that can be essential from the industrial point of view. *Pseudomonas putida* KT2440 has gained much attention in recent years as a metabolically versatile bacterium which has become an efficient cell factory for the production of value-added compounds [38]. Its versatility, degradative potential, and capacity to use a wide range of carbon sources make this bacterium an extraordinary candidate for industrial application. *P. putida* KT2440 has been used many times for mcl-PHAs production using different carbon sources. In addition, the molecular background of mcl-PHAs synthesis has been investigated in many studies so far, providing valuable information about the metabolic pathways leading to mcl-PHAs synthesis, the genes coding for the enzymes that are the engines of these conversion pathways (reviewed in [40]), and the molecular regulation of these transformations (reviewed in [28]). However, the overall picture of the molecular mechanisms related to mcl-PHAs synthesis is not clear. In particular, there is a lack of information about differences at the transcriptomic level when different carbon sources are used, and this knowledge could be useful for understanding the intricacies of carbon metabolism in *P. putida* KT2440 under nutrient starvation. Therefore, this study compared the transcriptomes of *P. putida* KT2440 during conversion of different carbon sources that require the use of either the de novo fatty acid synthesis pathway (sodium gluconate) or the β-oxidation pathway (oleic acid). The metabolic response of *P. putida* KT2440 to nitrogen-limiting conditions and a potential mechanism of global regulation are discussed.

## Materials and methods

### Growth conditions

*Pseudomonas putida* KT2440 (ATCC^®^ 47054™) was grown in a nitrogen-limited mineral medium consisting of (per liter): 2 g Na_2_HPO_4_·12H_2_O, 2 g KCl, 0.3 g Na_2_SO_4_, 1 g (NH_4_)SO_4_, 1 g MgSO_4_·7H_2_O, and 2.5 mL of trace element solution. Each liter of trace element solution contained: 20 g FeCl_3_·6H_2_O, 10 g CaCl_2_·H_2_O, 0.03 g CuSO_4_·5H_2_O, 0.05 g MnCl_2_·4H_2_O, and 0.1 g ZnSO_4_·7H_2_O dissolved in 0.5 N HCl. The cultures were supplemented with sodium gluconate or oleic acid as the only substrate in the same carbon concentration (3.8 g/L) in the production media. The cultivation was carried out in a 5 L working volume in a bioreactor (BioFlo 110, New Brunswick Scientific) at 30 °C with an aeration rate of 4 L/min. pH value was maintained at 7 through the modulated addition of concentrated 1 N NaOH and 1 N HCl. The dissolved oxygen was monitored during the whole cycle with O_2_ electrode (InPro 6800, Mettler Toledo GmbH, Switzerland) and maintained 50% air saturation by adjusting the agitation rate from 300 to 1000 rpm automatically. A concentrate solution (Sigma-Aldrich, USA) was used as antifoam in response to the antifoam controller. Total fermentation time was 48 h. In addition, parameters such as biomass, mcl-PHA, nitrogen, and phosphorus concentrations were controlled during the experiments.

### Analytical methods

The bacterial cell density was monitored as absorbance at 600 nm (OD_600_) with a spectrophotometer. During the fermentation, samples of the culture broth were taken for analysis at 8, 17, 24, 32, 41, and 48 h for measurements of cell dry weight, mcl-PHAs accumulation, and ammonium/phosphorus concentration, and for determination of monomers composition and their concentrations. Cell dry weight (CDW) was determined by centrifuging 100 mL culture broth at 11,200*×g* for 10 min. The collected cells were then weighed after lyophilization. The lyophilization process was performed by Lyovac GT2 System (SRK Systemtechnik GmbH) for 24 h. Ammonium and phosphorus concentration was measured spectrophotometrically using the Hach Lange DR 2800 spectrophotometer (Hach Lange, Düsseldorf DE) and the LCK303 kit for ammonium and LCK350 kit for phosphorus according to the manufacturer’s instructions.

Mcl-PHAs were extracted from lyophilized cells using the chloroform/methanol procedure for quantitative and qualitative analysis. These biopolyesters were extracted by shaking of the freeze-dried cells in chloroform at 50 °C for 3 h. The mixture was filtered through No. 1 Whatman filter paper by simple filtration and then precipitated with four volumes of 70% solution of chilled methanol and then allowed to evaporate for at room temperature. The monomeric composition of the purified mcl-PHAs was determined using a methanolysis protocol as described previously [33]. The concentrations of methyl esters were estimated by a gas chromatography (GC) equipped with a capillary column Varian VF-5 ms with a film thickness of 0.25 μm (Varian, Lake Forest, USA). Pure standards of methyl 3-hydroxyhexanoate, -octanoate, -nonanoate, -decanoate, -undecanoate, -dodecanoate, -tetradecanoate, and -hexadecanoate were used to generate calibration curves for the methanolysis assay. All samples were analyzed in triplicates.

### RNA extraction

Total RNA was extracted from the RNALater™-preserved bacterial cells using a commercial RNA extraction Kit (A&A Biotechnology, Poland) according to the manufacturer’s instructions. Total RNA was eluted with 50 μL of RNase-free water and contaminating DNA was removed via the On-Column DNase I Digest Set (Sigma-Aldrich, USA). Each time, the absence of contaminating DNA was proven by PCR reaction. The RNA concentration and purity were determined using a Bioanalyzer with the RNA 6000 Nano Assay (Agilent Technologies, Santa Clara, CA) according to the manufacturer’s protocol. The RNA integrity number (RIN) of every RNA sample used for sequencing was higher than 8.0. Samples for Illumina sequencing were collected for RNA isolation at 24 and 41 h of the fermentation, with each time corresponding to a different growth phase.

### Library preparation, Illumina sequencing, and RNAseq data analysis

RNAseq template libraries were constructed with 1 μg of the enriched mRNA samples using Truseq RNA Sample Preparation Kit (Illumina, California, USA) through a four-step protocol of end repairing, adding adenylate 3′ ends, adapter ligation, and cDNA template enrichment. Deep sequencing was performed by Illumina HiSeq 2500 according to the manufacturer’s instruction with a read length of 1 × 50 nucleotides. Raw data were pre-processed using FASTX-Toolkit (version 0.0.13) to remove low-quality sequences and adaptors. Clean RNAseq reads were aligned to the corresponding *Pseudomonas putida* KT2440 genome, which were revisited previously using Bowtie with the default parameters [4]. Genome sequences and annotation data of *P. putida* KT2440 were downloaded from NCBI (downloaded on 20 April, 2017). The reads that mapped to rRNA and tRNA sequences were excluded from further analysis. The reads per gene values of all genes were calculated from the SAM output files. Testing for differential expression was performed with DESeq package that uses a statistical model based on the negative binomial distribution [1]. Significant differential expressed genes with a false discovery rate (FDR value) of < 0.05 and a fold change of > 2 were selected for further analysis. The raw RNAseq data were deposited to the NCBI Sequence Read Archive (SRA) database with BioProject accession: SAMN06553737, SAMN06553738, SAMN06553739, and SAMN06553740.

### Real-time quantitative PCR (qPCR)

RNAseq data were confirmed by examining the expression of *phaC1*, *phaC2*, *phaZ*, *phaD*, *phaI*, *phaF,* and *phaG* genes by qRT-PCR. Total RNA was extracted as described above. The cDNA was performed using a SuperScript Vilo™ cDNA Synthesis Kit (Invitrogen) according to the manufacturer’s protocol. The synthesized first-strand cDNA was directly used as the template for qRT-PCR using SYBR Green technology in an ABI 7500 real-time PCR system (Applied Biosystems, USA) in MicroAmpTM Optical 96-well reaction plates (Applied Biosystems, USA). The primer pairs used for real-time amplification were described previously [36]. Standard curves using primer sets for *pha* genes were amplified against various concentrations of *Pseudomonas putida* KT2440 genomic DNA (from 5 × 10^−7^ to 5 ng). The reactions were run using the thermal cycling parameters as follows: 95 °C for 3 min, then 40 cycles of 95 °C for 15 s, and 60 °C for 1 min. After performing a run, a final standard melting curve stage was included. In each run, negative controls (no cDNA) for each primer set were included.

## Results

### Growth and mcl-PHAs synthesis

To show differences in mcl-PHAs synthesis by *Pseudomonas putida* KT2440, two fermentation batches, supplemented with metabolically different carbon sources, were conducted. The bacterial cells were grown in a nitrogen-limited mineral salt medium containing sodium gluconate or oleic acid as the only carbon source.

**Fig. 1:**
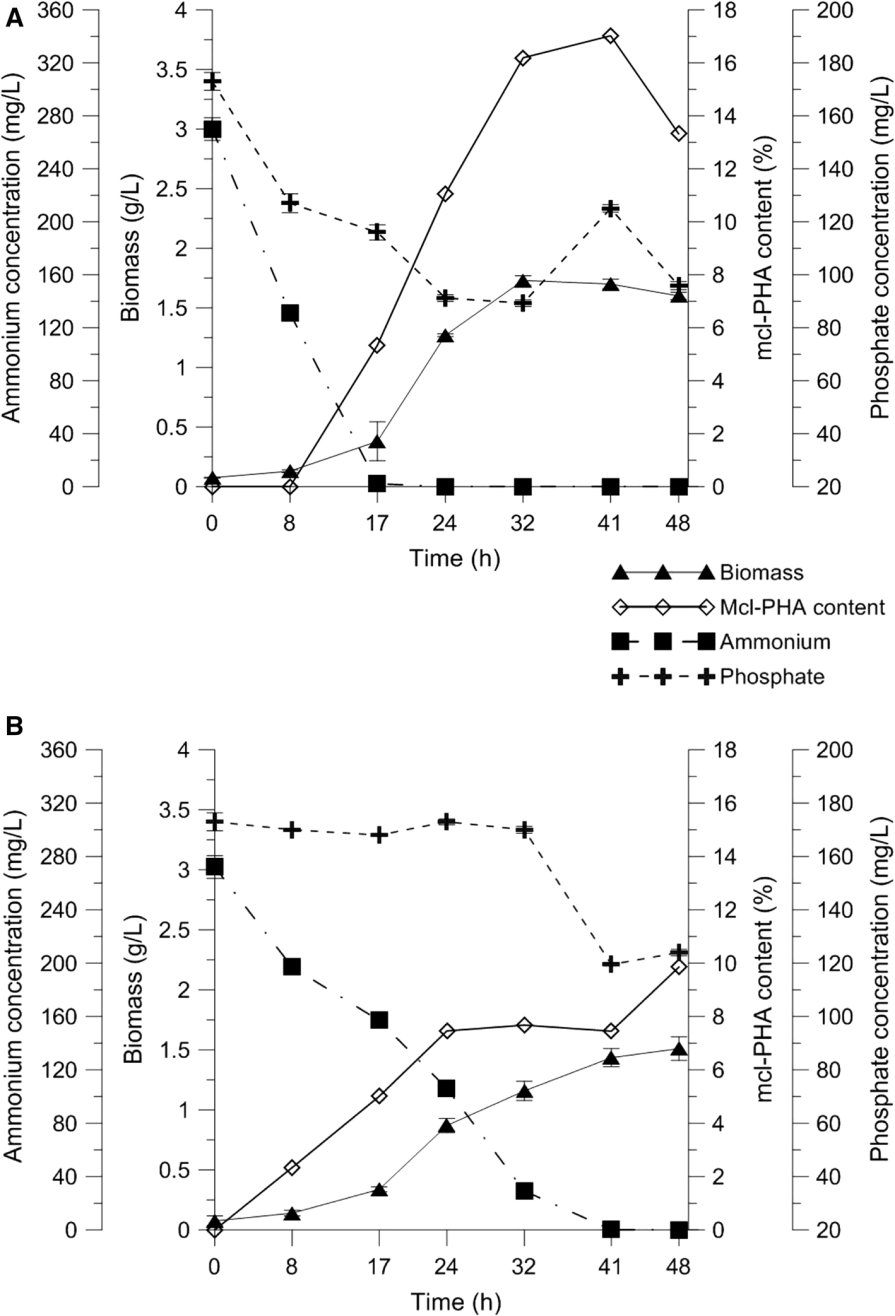
Growth mcl-PHAs and nutrient concentration during fermentation cultures of *P. putida* KT2440 grown on sodium gluconate (**a**) or oleic acid (**b**) as the only carbon sources

**Fig. 2:**
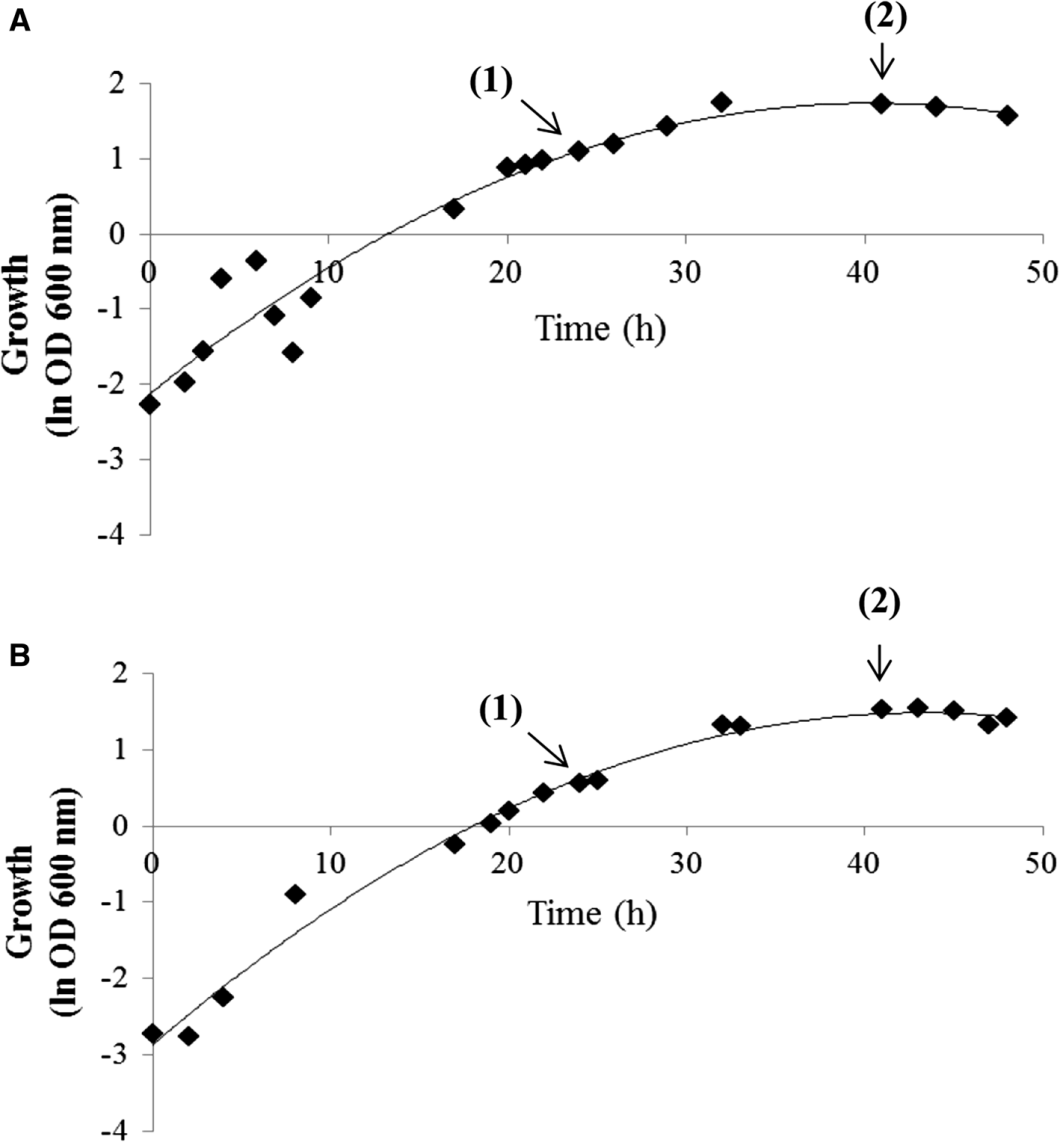
Growth during fermentation cultures of *Pseudomonas putida* KT2440 grown on sodium gluconate (**a**) or oleic acid (**b**). Numbers in parenthesis indicate the sampling time of biomass for total RNA isolation used for RNAseq analysis. (1) and (2) indicate samples collected at 24 and 41 h of the cultivation, respectively

As shown in Fig. 1, the *Pseudomonas putida* KT2440 cells initially grew without accumulating PHAs. The bacteria growing on sodium gluconate and oleic acid started to synthesize mcl-PHAs after 8 h of the fermentation. However, the level of mcl-PHAs in bacterial cells was higher with sodium gluconate. With sodium gluconate in the medium, ammonium was completely consumed during the first 17 h of the bioprocess. When the nitrogen source was depleted, the amount of mcl-PHAs value started to increase rapidly from 5.34% (at 17 h) to 17.02% (at 41 h) of cell dry weight (CDW). Then, the content of these biopolymers decreased, reaching 13.32% at the end of cultivation. *Pseudomonas putida* KT2440 accumulated less mcl-PHAs when cultivated on oleic acid (2.34% of CDW at 17 h and 9.86% of CDW at 48 h) than when cultivated on sodium gluconate. In the oleic acid variant, the bacteria took 41 h to deplete the entire amount of nitrogen. However, as initially desired, the biomass was similar in both cultivations. In both variants, at 24 h, *Pseudomonas putida* KT2440 was in the exponential phase and at 41 h of cultivation in the stationary phase (Fig. 2). In both experimental conditions, only the nitrogen stress was observed, and phosphate concentrations did not appear to be limiting factors. During both fermentations, phosphorus concentrations only slightly decreased throughout the entire process.

The composition of the extracted and purified mcl-PHAs was determined by GC of the 3-hydroxyalkanoates methyl esters. The biopolyesters was found to consist mainly of 3-hydroxyoctanoate and 3-hydroxydecanoate, lesser amounts of 3-hydroxydodecanoate, and trace amounts of 3-hydroxyhexanoate. 3-hydroxytetradecanoate was found in low amounts in mcl-PHAs obtained from *P. putida* KT2440 cultivated on sodium gluconate (Table 1). The major repeat units were similar to those produced by other *Pseudomonas* species cultivated on related and non-related carbon sources [9, 10].

**Table 1:** Monomeric composition of mcl-PHAs synthesized by *Pseudomonas putida* KT2440 grown on sodium gluconate and oleic acid

Culture time (h)	mcl-PHAs’ composition (mol%)									
	3HB	3HV	3HHx	3HO	3HN	3HD	3HUD	3HDD	3HTD	3HHxD
Sodium gluconate										
8	n.d.	n.d.	n.d.	n.d.	n.d.	n.d.	n.d.	n.d.	n.d.	n.d.
17	n.d.	n.d.	n.d.	n.d.	n.d.	82.6 ± 10.1	n.d.	17.4 ± 1.3	n.d.	n.d.
24	n.d.	n.d.	n.d.	14.3 ± 3.4	n.d.	75.4 ± 9.8	n.d.	8.6 ± 1.6	1.8 ± 0.2	n.d.
32	n.d.	n.d.	1.3 ± 0.1	18.3 ± 2.9	n.d.	71.9 ± 6.5	n.d.	7.1 ± 1.7	1.3 ± 0.3	n.d.
41	n.d.	n.d.	1.3 ± 0.2	19.8 ± 2.9	n.d.	71.0 ± 7.6	n.d.	6.5 ± 0.5	1.4 ± 0.3	n.d.
48	n.d.	n.d.	1.2 ± 0.1	19.5 ± 2.5	n.d.	71.2 ± 8.3	n.d.	6.7 ± 0.9	1.3 ± 0.3	n.d.
Oleic acid										
8	n.d.	n.d.	3.6 ± 0.0	38.9 ± 1.8	n.d.	37.2 ± 1.8	n.d.	20.4 ± 0.9	n.d.	n.d.
17	n.d.	n.d.	n.d.	32.7 ± 5.6	n.d.	44.8 ± 7.9	n.d.	22.5 ± 5.6	n.d.	n.d.
24	n.d.	n.d.	n.d.	42.6 ± 6.8	n.d.	39.4 ± 5.0	n.d.	18.1 ± 3.2	n.d.	n.d.
32	n.d.	n.d.	2.1 ± 0.2	38.8 ± 2.0	n.d.	39.2 ± 3.3	n.d.	19.9 ± 0.9	n.d.	n.d.
41	n.d.	n.d.	2.5 ± 1.3	36.6 ± 5.9	n.d.	41.1 ± 6.8	n.d.	19.9 ± 3.2	n.d.	n.d.
48	n.d.	n.d.	3.0 ± 0.6	32.8 ± 3.0	n.d.	41.4 ± 3.1	n.d.	22.9 ± 7.7	n.d.	n.d.

*n.d.* not detected, *3HB* 3-hydroxybutyrate, *3HV* 3-hydroxyvalerate, *3HHx* 3-hydroxyhexanoate, *3HO* 3-hydroxyoctanoate, *3HN* 3-hydroxynonanoate, *3HD* 3-hydroxydecanoate, *3HUD* 3-hydroxyundecanoate, *3HDD* 3-hydroxydodecanoate, *3HTD* 3-hydroxytetradecanoate, *3HHxD* 3-hydroxyhexadecanoate

### Expression levels of mcl-PHAs biosynthetic genes

**Fig. 3:**
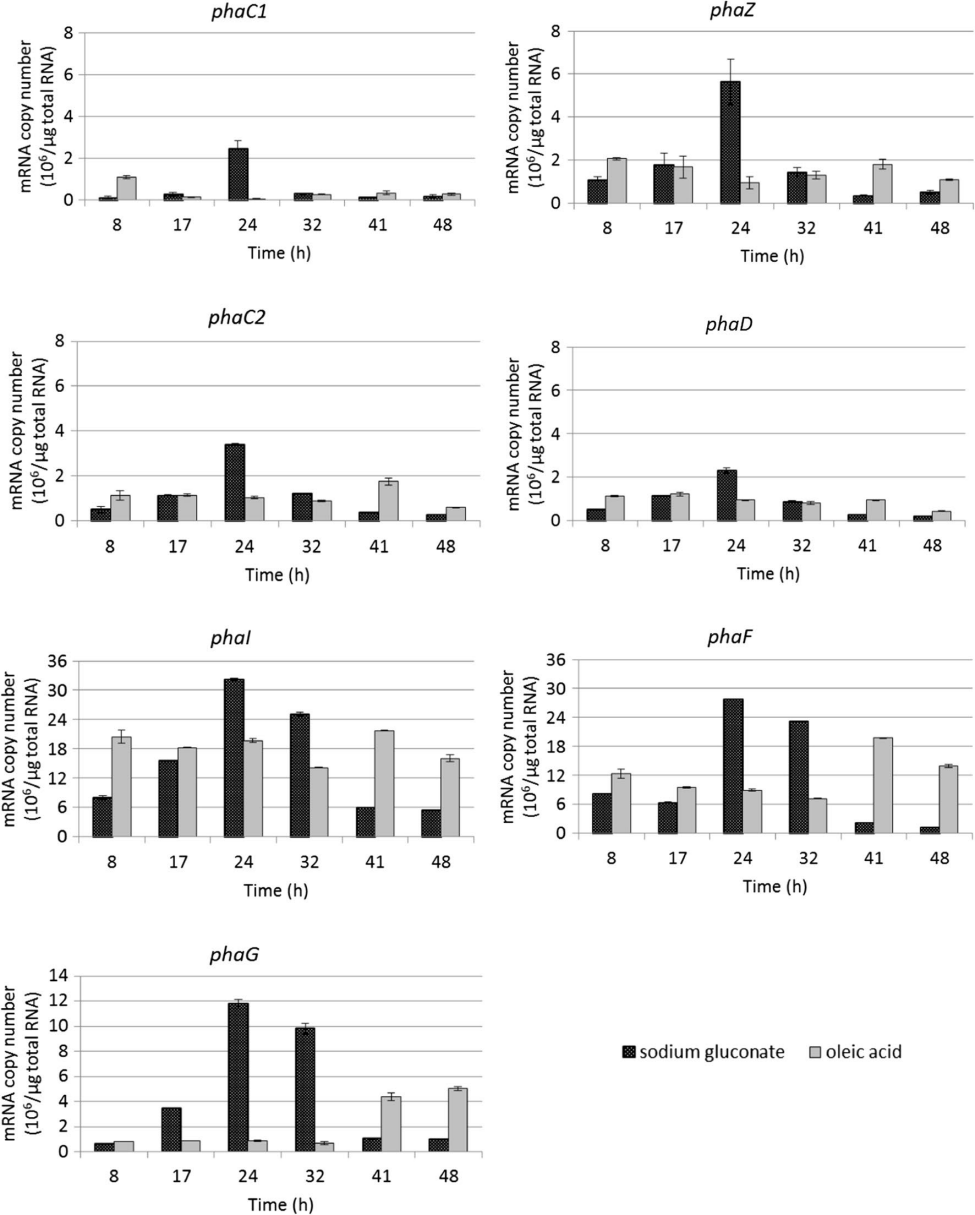
Quantitative RT real-time PCR analysis of *pha* genes. Each datum represents the mean ± standard deviation

The expression levels of *phaC1*, *phaC2*, *phaZ*, *phaD*, *phaI*, *phaF,* and *phaG* genes were monitored by reverse transcription real-time PCR. As can be seen in Fig. 3, the mRNA copy numbers differed between the types of substrate. During the fermentation with sodium gluconate, the transcript numbers of the investigated genes were highest at 24 h of fermentation, when ammonium was completely consumed. After this, they declined up until the end of fermentation. When oleic acid was used, the nitrogen depletion (after 32 h) induced mainly the expression of *phaF* and *phaG* genes. With both carbon sources, the transcript numbers of the *phaI* and *phaF* genes were much higher than those of the other genes involved in mcl-PHAs synthesis. The profile of *phaF*, *phaI,* and *phaG* expression differed depending on the substrate that was used. With sodium gluconate, their expression was higher at 24 and 32 h than at other times. With oleic acid, the expression of these genes reached the highest level at 41 h. With sodium gluconate, the changes in transcription over time of all *pha* genes were similar. Furthermore, during the fermentation with sodium gluconate, all *pha* genes had lower expression at 41 h than at 24 h, whereas these genes had higher expression with oleic acid at that time period.

### Overview of transcriptomic analysis

A total of about 150-million raw sequencing reads were generated from RNAseq data using the Illumina platform, with an average of 37.5 million reads per sample. After the elimination of low-quality reads with multiple N, reads shorter than 20 bp, and removal of sequences coding for rRNA, a total of 119 million qualified mRNA sequence reads were mapped onto the *Pseudomonas putida* KT2440 genome. After expression quantification, differential gene expression analysis was carried out between samples collected at 24 and 41 h of the cultivation with both carbon sources, as these time corresponded to a different cellular phases (exponential and stationary). The expression levels for each gene were quantified as reads per kilobase per million mapped reads (RPKM), as described by Mortazavi et al. [32].

**Fig. 4:**
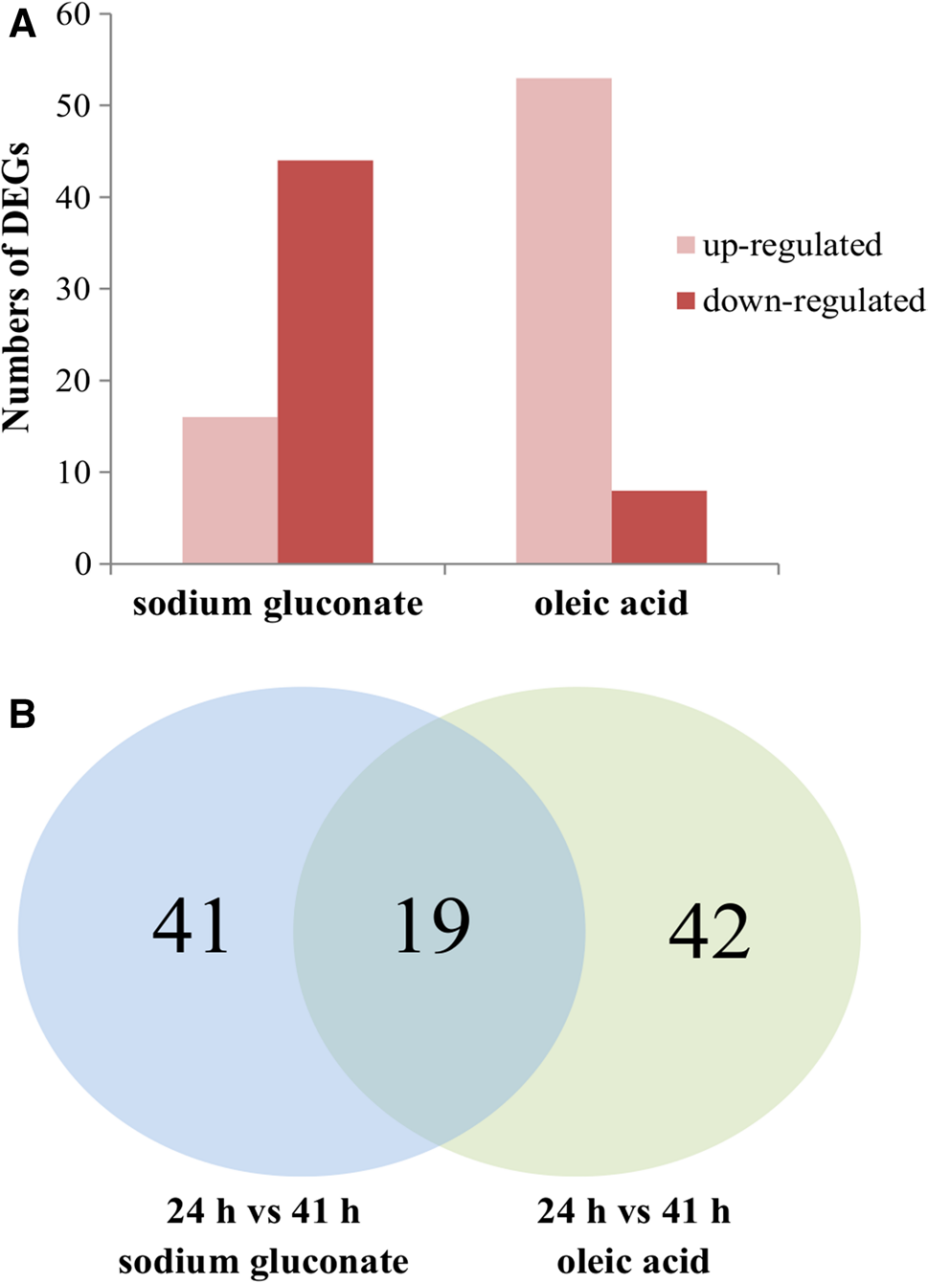
Number of significantly differentially expressed genes in bacterial cells growing on sodium gluconate and oleic acid towards mcl-PHAs synthesis. **a** Number of up-regulated and down-regulated genes between samples at 24 and 41 h of the cultivation. **b** Venn diagram showing the numbers of common and specific DEGs in samples with metabolically different substrates

The DESeq analysis led to identification of 60 and 61 genes for sodium gluconate and oleic acid, respectively, whose expression differed significantly (*p* < 0.05) between 24 and 41 h. With sodium gluconate, 16 differentially expressed genes were up-regulated with fold changes ranging from 6.2 to 65.3 (Supplementary material S1). With oleic acid, interestingly, most genes (53 genes) were up-regulated with fold changes from 4.7 to 818.8 (Supplementary material S2). In general, 19 genes were significantly differentially expressed at both analyzed time-points in both cultivations, while 41 genes and 42 genes were specifically induced in *Pseudomonas putida* KT2440 cultivated on sodium gluconate or oleic acid, respectively (Fig. 4).

**Fig. 5:**
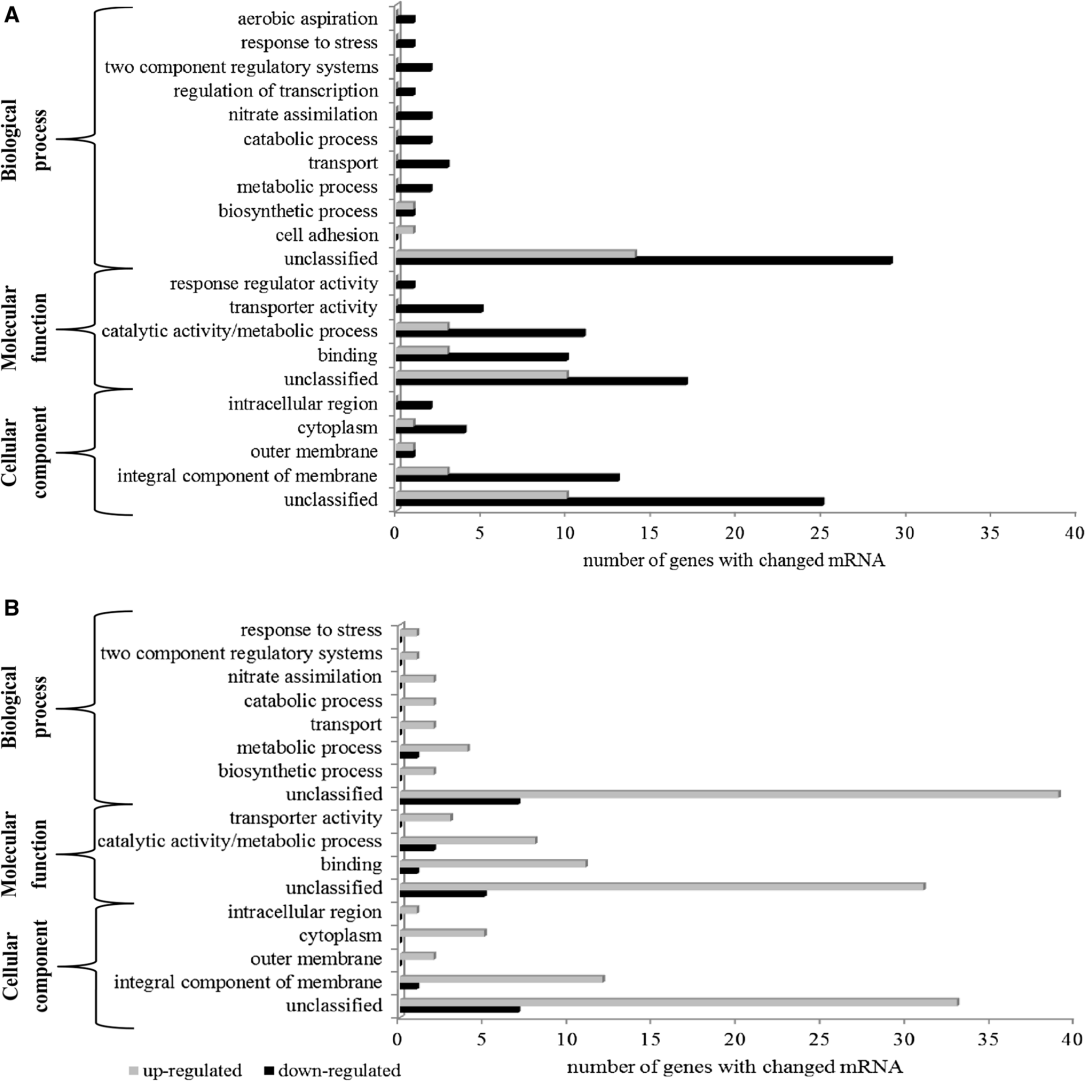
Gene ontology analysis of the significantly differentially expressed genes between 24 and 41 h of the cultivation of *P. putida* KT2440 grown on sodium gluconate (**a**) or oleic acid (**b**)

The RNAseq data provide detailed information about the genes that are regulated (induced or suppressed) in response to nitrogen starvation during mcl-PHAs synthesis. The differentially expressed genes were sorted into role categories to evaluate which functions could be related to mcl-PHAs biosynthesis process during cultivation on the two different carbon sources (sodium gluconate or oleic acid). In the biological ontology, we found that the most abundant terms were transport and metabolic processes. In the cellular component, integral component of membrane was the dominant term with both substrates, and in the molecular function ontology, binding and catalytic activity terms were dominant (Fig. 5). Furthermore, pathway analyses were used to identify the significant pathways associated with the differentially expressed genes according to KEGG. With sodium gluconate, the up-regulated genes were mainly involved in the citrate cycle (TCA cycle), glycolysis/gluconeogenesis, pyruvate metabolism, microbial metabolism in diverse environments, biosynthesis of secondary metabolites, and carbon metabolism. In particular, the transcript levels of genes encoded by PP_2858, PP_2855, PP_4057, and those involved in acetoin metabolism (*acoA*, *acoB*, and *acoC*) were elevated, whereas those involved in the pentose-phosphate pathway, ABC transporters, nitrogen metabolism, and two-component systems were down-regulated. The gene encoding Xaa-Pro aminopeptidase (PP_4752), syringomycin biosynthesis protein 2 (PP_3783), and gluconate 2-dehydrogenase gamma subunit (PP_3384) were the most significantly down-regulated. When cultivations were supplemented with oleic acid, the up-regulated genes of *Pseudomonas putida* KT2440 were found to be involved in nitrogen metabolism, arginine biosynthesis, microbial metabolism in diverse environments, purine metabolism, and ABC transporters. The pathways in which the down-regulated genes could be involved were not identified; for the most part, these genes encode hypothetical proteins. With both carbon sources, the following genes were significantly differentially expressed during mcl-PHAs synthesis: genes involved in nitrogen metabolism (*nirB*, *nirD*, and *nasA*), microbial metabolism in diverse environments (*ureA* and *ureB*), ABC transporters (*urtB*, *urtC*, and *urtD*), and two-component system response regulator NasT (PP_2093).

**Fig. 6:**
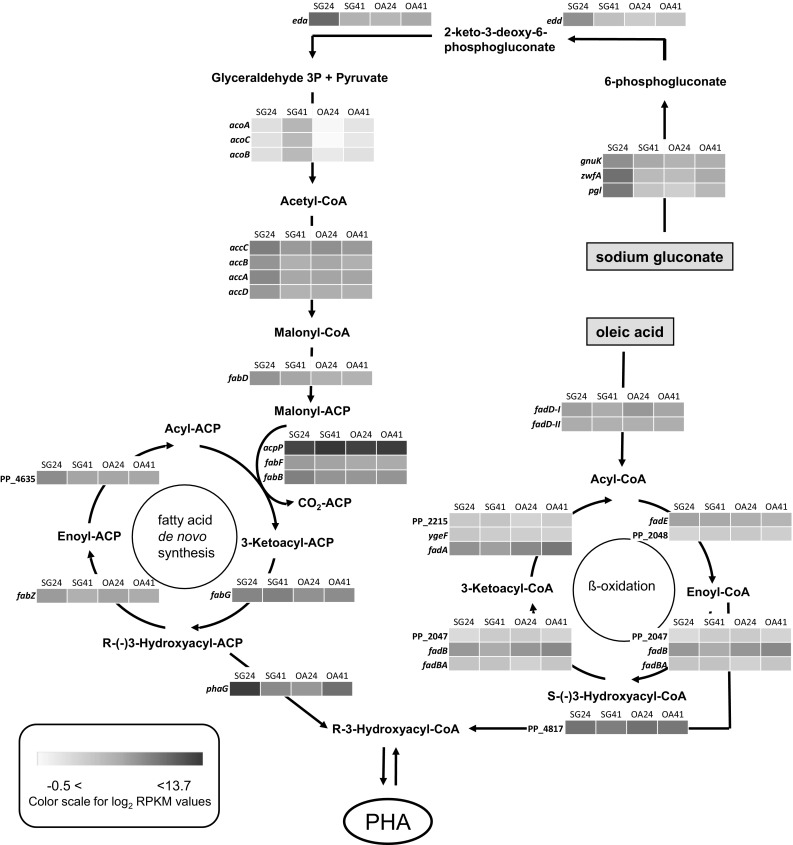
Changes in the expression of genes involved in central pathways for (*R*)-3-hydroxyacyl-CoA generation in *P. putida* KT2440 affected by growth on sodium gluconate or oleic acid under nitrogen stressful conditions. *SG24*-sodium gluconate‚ 24 h of the cultivation; *SG41*-sodium gluconate‚ 41 h of the cultivation; *OA24*-oleic acid‚ 24 h of the cultivation; *OA41*-oleic acid‚ 41 h of the cultivation

With regard to carbon source metabolism (Fig. 6), the data suggest that the expression level of genes involved in the Entner–Doudoroff pathway, pyruvate metabolism, fatty acid de novo synthesis, and β-oxidation pathway could have an effect on increased mcl-PHAs synthesis. *Pseudomonas putida* KT2440 grown on sodium gluconate accumulated about 1.5 times more mcl-PHAs at 41 h of the fermentation than at 24 h. During this cultivation, all genes engaged in above-mentioned metabolic pathways had lower expression at 41 h than at 24 h, except for the *acoA*, *acoB*, and *acoC* genes, which were significantly up-regulated. To evaluate the effect of these genes on biopolymers production, we collected samples at the same time-points from the bioreactor supplemented with oleic acid. In this cultivation, mcl-PHAs concentration remained the same (about 7% of CDW at 24 and 41 h). In addition, expression of the *acoA*, *acoB*, and *acoC* genes did not change significantly. Furthermore, the expression of genes related to the synthesis of malonyl-CoA from acetyl-CoA seems to be dependent on the carbon source that is used. At 24 h, the expression of *accA*, *accB*, *accC*, and *accD* genes was the highest in *P. putida* KT2440 cultivated on sodium gluconate, and these values were about threefold higher than in the same culture at 41 h. A similar trend was noted during cultivation with oleic acid, although the decrease in expression over time was smaller, especially for *accB* and *accD* gene, which were 1.5-fold and 0.1-fold lower at 41 h, respectively. When comparing the two substrates, the expression of each of these genes at each time point was at least threefold lower with sodium gluconate than with oleic acid. The genes *fabF*, *fabB*, *fabZ*, and PP_4635, which are involved in de novo fatty acid synthesis, were highly expressed at 24 h, except for *fabG* and *acpP* which expression was on the same or slightly higher level at 41 h compared to the growth phase. Furthermore, the genes related to β-oxidation pathway were expressed also in the cultivation with sodium gluconate as the only substrate. Their expression levels were similar to the transcripts abundance observed in the fermentation with oleic acid.

### Validation of Illumina sequence data using qRT-PCR

To confirm the data obtained by RNAseq, the expression of the most important genes for mcl-PHAs synthesis (*phaC1*, *phaZ*, *phaC2*, *phaD*, *phaI*, *phaF,* and *phaG*) was determined with real-time quantitative PCR (qPCR). The qPCR results of the majority of analyzed genes were in good agreement with the RNAseq data indicating the high credibility of these genes in transcript abundance suggesting a good quality of RNAseq data. In a case of *phaC1*, *phaI,* and *phaF* genes, the discrepancies in the fold-change level between RNAseq and qPCR were observed, but still the trend in the changes of their expression profiles was the same (Table 2).

**Table 2:** Validation of RNAseq data by RT-qPCR analysis

ID	Gene	Description	Sodium gluconate			Oleic acid		
			24 vs 41 h		Change	24 vs 41 h		Change
			RNAseq	RT		RNAseq	RT	
PP_5003	*phaC1*	PHA polymerase	− 3.28	− 4.19	Down	0.93	2.59	Up
PP_5004	*phaZ*	PHA depolymerase	− 2.57	− 3.99	Down	1.51	0.93	Up
PP_5005	*phaC2*	PHA polymerase	− 2.76	− 3.21	Down	1.19	0.74	Up
PP_5006	*phaD*	Transcriptional regulator	− 2.41	− 3.14	Down	0.32	0.00	Up
PP_5007	*phaI*	PHA granule-associated	− 5.15	− 2.42	Down	2.63	0.14	Up
PP_5008	*phaF*	PHA granule-associated	− 3.29	− 3.69	Down	0.25	1.15	Up
PP_1408	*phaG*	Acyl-transferase	− 4.85	− 3.52	Down	2.48	2.28	Up

*RT* RT-qPCR data

## Discussion

Bacterial adaptation to nutrient limitation is of interest not only because it enables the bacteria to survive in harsh environments, but also because it can induce valuable bioproducts’ production. The mechanism involved in the regulation of mcl-PHAs synthesis at the transcriptional level seems to be important, and thus, great effort is being made to detect genes potentially involved in this bioprocess. The investigation of molecular mechanisms that drive mcl-PHAs synthesis may provide valuable information to improve the efficiency of this bioprocess and make it more economically feasible.

This study used RNAseq technologies to examine and compare the transcriptomes of *Pseudomonas putida* KT2440 cultivated on two metabolically different carbon sources. In general, the presented results indicated the difference in the transcriptional response to nitrogen limitation during microbial mcl-PHAs synthesis. Although *Pseudomonas putida* KT2440 has been used as a model microorganism in different studies including those looking at molecular machinery [5, 8, 13, 39], its nitrogen and substrate response transcriptome has not yet been analyzed and compared. To better understand the process, a time course study of the *Pseudomonas putida* KT2440 response transcriptome was carried out.

The data confirm that *Pseudomonas putida* KT2440 was able to synthesis mcl-PHAs under the applied conditions. Among *pha* cluster genes, *phaF and phaI* genes showed the highest expression levels and the trend of their expression changes was similar. It has been suggested that PhaF and PhaI phasins play a crucial role in PHAs granule formation and localization [15]. In our study, the highest mcl-PHAs content in bacterial cells growing on both substrates was proceeded by the highest levels of *phaF* and *phaI* transcripts. It could be suggested that the overproduction of *pha* genes, especially PhaI and PhaF phasins, could improve mcl-PHAs biosynthesis process. Similar behaviour in transcription showed *phaG* gene coding for 3-hydroxyacyl-ACP:CoA transacylase-linking fatty acids de novo synthesis and biosynthesis of medium-chain-length polyhydroxyalkanoates [42, 44]. PhaG was reported to be important for synthesizing mcl-PHAs from non-related carbon sources such as gluconate and glucose [19]. Our results add to these findings by showing that *phaG* expression is associated with mcl-PHAs synthesis on oleic acid (a structurally related carbon source), suggesting that PhaG may play a role in this process, as well. An association between increased *phaG* expression and mcl-PHAs synthesis on oleic acid was also found in our previous work using the *P. putida* KT2440 *relA/spoT* mutant [36]. Our results also confirm that the expression level of *pha* cluster genes and the concentration of mcl-PHAs that are accumulated are strongly correlated.

There are several metabolic routes that could be involved in the synthesis of 3-hydroxyacyl-CoA thioesters. On non-related carbon sources like gluconate or glycerol, fatty acid de novo synthesis is active during growth, but when a cultivation is supplemented with fatty acids as a substrate, β-oxidation is the main pathway. The first step of fatty acid biosynthesis, the synthesis of malonyl-CoA from acetyl-CoA, is catalyzed by acetyl-CoA carboxylase (ACC). ACC is composed of four distinct proteins; biotin carboxyl carrier protein (AccB), biotin carboxylase (AccC), and two subunits each of ACCase subunit alpha (AccA) and ACCase subunit beta (AccD). The conversion of acetyl-CoA to malonyl-CoA takes place in distinct partial reactions. The first step is the transfer of CO_2_ in an ATP-dependent fashion from bicarbonate to the biotin moiety of AccB, resulting in carboxybiotinoyl-AccB. In the second step, the CO_2_ group is subsequently transferred from carboxybiotin to acetyl-CoA to form malonyl-CoA [11]. Since acetyl-CoA carboxylase catalyzes the initial step of fatty acid biosynthesis, it has been the subject of very intensive study. In particular, ACC could be important in the PHA synthesis process [22, 44]. In our study, RNAseq analysis revealed that the expression levels of *accA*, *accB*, *accC,* and *accD* genes were high in the growth phase and then decreased in the stationary phase; however, their transcripts levels were still high in the PHAs production phase, suggesting that the regulation of all four acetyl-CoA carboxylase genes depends on the phase of growth, and that these genes may influence mcl-PHAs concentration. The *Pseudomonas putida* cells growing at a slower rate on oleic acid (the biomass value reached from 0.87 to 1.4 g/L) synthesized 2.3-times less mcl-PHAs than those growing on sodium gluconate. In both cultures, the level of *accABCD*-specific mRNA decreased as the bacteria entered the stationary phase. The Acc cluster has been demonstrated to regulate growth rate in *Bacillus subtilis* and *E. coli* [23], in which the *accBC* genes behave analogously to those genes in the *Pseudomonas putida* KT2440 in our study. In addition, overexpression of Acc (encoding *E. coli* ACC) has been reported to improve the free fatty acids titer to 0.81 g/L in a batch fermentation [27], indicating that these proteins play an important role in the fatty acids de novo synthesis pathway. Our results suggest that the genes coding for acetyl-CoA carboxylase could coordinate bacterial growth and mcl-PHAs biosynthesis. It could be hypothesized that overexpressing of the genes coding for acetyl-CoA carboxylase may increase the rate of polyhydroxyalkanoates biosynthesis in *P. putida* KT2440.

Furthermore, the RNAseq revealed that genes involved in the β-oxidation pathway were expressed not only during cultivation on oleic acid but also on sodium gluconate, as shown in Fig. 6. The expression levels of these genes on both substrates were similar. A previous study on *Ralstonia eutropha* H16 also suggested that the fatty acid β-oxidation pathway was functional in the presence of a non-related carbon source [44]. Fatty acid synthesis may stop when a certain chain length is reached, after which acyl-ACP, which can be converted to acyl-CoA via the β-oxidation pathway, is used for formation of membrane lipids [24]. We predict that FadD (an acyl-CoA synthetase) activated free fatty acids released from membrane lipids into acyl-CoA, and then, they were degraded by β-oxidation pathway.

Previously, it had been reported that the Entner–Doudoroff (ED) pathway seems to be essential for *Pseudomonas* to metabolize glucose or gluconate [12]. External gluconate can be converted to 6-phosphogluconate and then either decarboxylated to pentose-phosphate, or metabolized to pyruvate and glyceraldehyde 3-phosphate via the ED route. The latter pathway involves two genes: *edd* (PP_1010) and *eda* (PP_1021), which encode gluconate 6-P dehydrogenase and 2-keto-3-deoxygluconate-6-P aldolase, respectively. Our results confirm that the *edd* and *eda* genes are also active when *Pseudomonas putida* KT2440 is cultivated on oleic acid. This finding suggests that the ED pathway is functional during mcl-PHAs production and plays a role when fatty acids are used as the only carbon source. This leads us to suggest that the ED pathway may play an important role in the physiology of *P. putida* KT2440 under environmental stress caused by nitrogen limitation. RNAseq analysis confirmed that the expression level of *edd* and *eda* genes at 24 h of the cultivations was higher with sodium gluconate as compared with oleic acid. However, in the stationary growth phase, the *eda* transcripts were higher when bacteria grown on oleic acid than on gluconate. It is worth noting that the rate of nitrogen consumption was much slower with oleic acid than with gluconate. Furthermore, the ED route may be essential in energy metabolism to provide an appropriate level of redox cofactors in balancing cellular reducing equivalents such as NADH/NADPH. The active ED pathway is not only necessary for the metabolizing of carbon sources but also to fulfilling the bacterial demands for NADPH [8, 37]. The more NADPH, the higher confactor availability [3], leading to an improvement of mcl-PHA synthesis rate. Furthermore, ED pathway works in combination with pentose-phosphate pathway (PP pathway) to synthesize pyruvate and acetyl-CoA. Previously, it was proven that the overexpression of PP pathway’s genes could not increase the PHAs productivity in *P. putida* KT2440 growing on glucose [5]. Nevertheless, we strongly point out that the genes of PP and ED pathways could be potential molecular targets affecting the cellular mcl-PHA biosynthesis. Moreover, the genes encoding the multicomponent acetoin dehydrogenase enzyme complex (AcoABC and PP_0556) were significantly up-regulated only on sodium gluconate. They catalyze the oxidative decarboxylation of pyruvate with the formation of acetyl-CoA, CO_2_, and NADH (H +). Based on our results, we hypothesize that the significant increase in *aco* transcripts had a positive effect on the efficiency of mcl-PHAs synthesis by increasing the pool of cellular acetyl-CoA and NADPH, which are used in fatty acid de novo synthesis during the phase of mcl-PHAs accumulation. The ratios of reducing equivalents are essential for PHAs biosynthesis in bacteria [43]. The overexpression of pyruvate dehydrogenase subunits in *P. putida* KT2440 grown on glucose, postulating that it is an important step in supplying NADH, and in a consequence, in maximizing mcl-PHAs production [14].

Aside from genes related to metabolism, a number of transporters were differentially regulated during mcl-PHAs synthesis. All of these genes were found to be down-regulated on sodium gluconate, and up-regulated on oleic acid. Although the expression of these genes was higher in the exponential growth phase than in the stationary phase, the level of expression was sufficient for biopolymer production. Nitrogen limitation induced the expression of an LysE family transporter (PP_2388). Its up-regulation in the bacterial cells growing on oleic acid may have supported the survival of these bacteria under stressful conditions. Members of the LysE superfamily have been reported to be involved in ionic homeostasis, protection from excessive metabolite concentrations, cell envelope assembly, and transmembrane electron flow [47]. The Major Facilitator Superfamily Transporter (MFS), which we found to have a higher expression in the exponential phase on sodium gluconate, also seems to play a role in mcl-PHAs synthesis. This is considered to be one of the largest classes of secondary active carriers, which allow the selective transport of a vast range of diverse substrates across the membrane [26].

Global transcriptomics revealed that, during cultivation on sodium gluconate, two regulatory genes (*ntrC* and PP_2093) were also differentially expressed in the transcriptome of *P. putida* KT2440, whereas during cultivation on oleic acid, only PP_2093 was observed to be significantly up-regulated. NtrC is a nitrogen-mediated regulator that controls a number of operons involved in the uptake and/or assimilation of alternative nitrogen sources when preferred nitrogen compound are scarce [17, 18]. In addition, NasT regulatory system (PP_2093) has been proposed to induce the nitrate/nitrite assimilatory pathway (generally *nas* genes), as it is widely distributed in bacteria that can assimilate NO_3_^−^/NO_2_^−^ as their sole source of nitrogen for growth when ammonium is limited [30]. The expression of a cytoplasmic assimilatory NO_3_^−^/NO_2_^−^ reductase system causes NO_3_^−^ to be converted to NO_2_^−^ and then to NH_4_^+^ [16]. After that, the ammonium is incorporated into glutamate and glutamine which donate nitrogen for biosynthetic cellular metabolism. Under the nitrogen-deficient growth conditions in our study, we postulate that the bulk of L-glutamate was formed by the concerted action of NH_4_-dependent glutamine synthetase and the glutamine: 2- oxoglutarate amidotransferase (also known as glutamate synthase) in the glutamine synthetase/glutamine:2-oxoglutarate amidotransferase cycle [31]. In our study, the expression of PP_2093 gene, encoding two-component system response regulator NasT, seems to be highest at the beginning of NH_4_ deficiency in the culture medium. This allowed the bacterial cells to respond immediately to changes in ammonium concentrations. In cultivation on sodium gluconate, *nasT* was overexpressed at 24 h, when NH_4_^+^ was depleted, whereas, on oleic acid, its expression level was lower at that time of the process, because NH_4_^+^ was not depleted until the end of cultivation.

In conclusion, a global transcriptomic approach during mcl-PHAs synthesis shows that the environmental stress leads to significant changes in the pattern of gene expression of the *Pseudomonas putida* KT2440 cells. The results confirm that the biopolymers concentration affects not only PHA synthesis-related genes but also the genes involved in crucial pathways giving the precursors for its biosynthesis. Surprisingly, our results revealed that *phaG* gene could be associated with the mcl-PHAs synthesis process not only on sodium gluconate but also on oleic acid. A comparison of transcriptomes also revealed that the genes involved in the ED pathway and those coding for acetyl-CoA carboxylase affect biopolymers accumulation. According to the transcriptomic data, several genes related to β-oxidation pathway were active both in growth and PHAs production phase even when *Pseudomonas* cells were cultivated on sodium gluconate. Furthermore, the expression of genes responsible for intracellular energy supply was modulated to fulfill the bacterial demands for redox cofactors in balancing cellular reducing equivalents. The results indicate that the above-mentioned genes could be possible targets in improving the mcl-PHAs production process. Current knowledge of regulatory machinery governing mcl-PHAs synthesis in *Pseudomonas putida* strains is limited. An understanding of the regulatory mechanisms can be achieved with the help of systems biology and functional proteomics.

## Electronic supplementary material

Below is the link to the electronic supplementary material.

**Supplementary material S1.** Significantly differentially expressed genes of *Pseudomonas putida* KT2440 grown on sodium gluconate. (PDF 115 kb)

**Supplementary material S2.** Significantly differentially expressed genes of *Pseudomonas putida* KT2440 grown on oleic acid. (PDF 112 kb)
