# Supplementary material for: Transcriptome remodeling of Pseudomonas putida KT2440 during mcl-PHAs synthesis: effect of different carbon sources and response to nitrogen stress
Source: J Ind Microbiol Biotechnol. 2018 May 7;45(6):433–46. doi: 10.1007/s10295-018-2042-4 (PMC6028892; doi:10.1007/s10295-018-2042-4)
Supplement: Supplementary file 1 — Supplementary material S1. Significantly differentially expressed genes of Pseudomonas putida KT2440 grown on sodium gluconate. (PDF 115 kb) [file 10295_2018_2042_MOESM1_ESM.pdf]

Journal of Industrial Microbiology & Biotechnology

**Transcriptome remodeling of *Pseudomonas putida* KT2440 during mcl-PHAs synthesis: effect of different carbon sources and response to nitrogen stress**

Justyna Mozejko-Ciesielska[1], Tomasz Pokoj[2], Slawomir Ciesielski[2]

**Correspondence:** Justyna Możejko-Ciesielska, Department of Microbiology, Faculty of Biology and Biotechnology, University of Warmia and Mazury in Olsztyn, Oczapowskiego 1A, 10-719 Olsztyn, e-mail: justyna.mozejko@uwm.edu.pl

**Supplementary material S1.** Significantly differentially expressed genes of *Pseudomonas putida* KT2440 grown on sodium gluconate

| Locus tag | Gene name | Description | Fold Change | $\log_2$Fold Change |
|---|---|---|---|---|
| PP_2858 | no_symbol | hypothetical protein | 65.2638 | 6.028 |
| PP_2855 | no_symbol | hypothetical protein | 31.641 | 4.983 |
| PP_4057 | no_symbol | membrane protein | 18.034 | 4.172 |
| PP_5428 | no_symbol | hypothetical protein | 13.836 | 3.790 |
| PP_0135 | no_symbol | hypothetical protein | 13.557 | 3.761 |
| PP_0555 | *acoA* | acetoin:2,6-dichlorophenolindophenol oxidoreductase subunit alpha | 13.237 | 3.726 |
| PP_0553 | *acoC* | etoin cleaving system dihydrolipoyllysine-residue acetyltransferase | 12.139 | 3.601 |
| PP_0554 | *acoB* | acetoin:2,6-dichlorophenolindophenol oxidoreductase subunit beta | 11.062 | 3.467 |
| PP_0556 | no_symbol | acetoin catabolism protein | 9.298 | 3.217 |
| PP_2719 | no_symbol | hypothetical protein | 8.121 | 3.021 |
| PP_3747 | *glcF* | glycolate oxidase iron-sulfur subunit | 8.085 | 3.015 |
| PP_3317 | no_symbol | hypothetical protein | 7.666 | 2.938 |
| PP_3065 | *gpD* | tail formation | 7.371 | 2.881 |
| PP_3542 | no_symbol | hypothetical protein | 7.138 | 2.835 |
| PP_4317 | no_symbol | hypothetical protein | 7.032 | 2.813 |
| PP_5560 | no_symbol | hypothetical protein | 6.230 | 2.639 |
| PP_4752 | no_symbol | Xaa-Pro aminopeptidase | 13.967 | -2.764 |
| PP_3783 | *syrB* | syringomycin biosynthesis protein 2 | 14.207 | -2.789 |
| PP_3384 | no_symbol | gluconate 2-dehydrogenase gamma subunit | 14.617 | -2.830 |
| PP_1788 | no_symbol | hypothetical protein | 15.032 | -2.870 |

| | | | | |
|---|---|---|---|---|
| PP_4751 | no_symbol | amino acid ABC transporter ATP-binding protein | 15.035 | -2.870 |
| PP_2846 | *ureE* | urease accessory protein | 15.125 | -2.879 |
| PP_3210 | no_symbol | ABC transporter permease | 15.333 | -2.899 |
| PP_2092 | *nasA* | nitrate transporter | 15.733 | -2.936 |
| PP_3782 | no_symbol | hypothetical protein | 15.888 | -2.950 |
| PP_3383 | no_symbol | gluconate 2-dehydrogenase flavoprotein subunit | 16.788 | -3.029 |
| PP_4845 | *urtE* | ABC transporter ATP-binding protein | 17.288 | -3.072 |
| PP_3382 | no_symbol | gluconate 2-dehydrogenase cytochrome c subunit | 17.700 | -3.106 |
| PP_0503 | no_symbol | MFS transporter | 17.770 | -3.111 |
| PP_2847 | *ureJ* | urease accessory protein UreJ | 18.312 | -3.155 |
| PP_2843 | *ureA* | urease subunit gamma | 18.571 | -3.175 |
| PP_3212 | no_symbol | Rieske 2Fe-2S family protein | 20.173 | -3.294 |
| PP_5390 | no_symbol | hypothetical protein | 20.919 | -3.347 |
| PP_1706 | *nirD* | nitrite reductase | 21.437 | -3.382 |
| PP_3214 | no_symbol | hypothetical protein | 23.950 | -3.542 |
| PP_3374 | no_symbol | hypothetical protein | 24.125 | -3.552 |
| PP_2387 | no_symbol | hypothetical protein | 24.395 | -3.568 |
| PP_1705 | *nirB* | nitrite reductase large subunit | 25.771 | -3.648 |
| PP_3375 | *endA* | endonuclease I | 27.032 | -3.717 |
| PP_2844 | *ureB* | urease subunit beta | 28.700 | -3.803 |
| PP_2388 | no_symbol | LysE family transporter | 29.365 | -3.836 |
| PP_4310 | no_symbol | hydantoin racemase | 30.037 | -3.869 |
| PP_1299 | *yhdY* | amino acid ABC transporter permease | 30.044 | -3.869 |
| PP_5048 | *glnG* | two-component system DNA-binding response regulator GlnL/GlnG | 32.083 | -3.964 |
| PP_2093 | no_symbol | two-component system response regulator NasT | 34.152 | -4.054 |
| PP_4252 | *ccoQ-I* | cbb3-type cytochrome c oxidase subunit | 34.333 | -4.062 |
| PP_4264 | *hemN* | oxygen-independent coproporphyrinogen III dehydrogenase | 34.500 | -4.068 |
| PP_2648 | no_symbol | universal stress protein family protein | 36.719 | -4.158 |

| | | | | |
|---|---|---|---|---|
| PP_2687 | no_symbol | hypothetical protein | 39.553 | -4.266 |
| PP_4251 | *ccoO-I* | cbb3-type cytochrome c oxidase subunit | 40.000 | -4.282 |
| PP_4843 | *urtC* | urea ABC transporter permease | 41.196 | -4.324 |
| PP_2686 | no_symbol | transglutaminase domain-containing protein | 42.437 | -4.367 |
| PP_4842 | *urtB* | urea ABC transporter permease | 43.403 | -4.400 |
| PP_2389 | no_symbol | hypothetical protein | 45.512 | -4.468 |
| PP_3378 | *kguK* | 2-ketogluconokinase | 51.891 | -4.657 |
| PP_4626 | *cidA* | murein hydrolases holin regulator | 60.955 | -4.890 |
| PP_1023 | *pgl* | 6-phosphogluconolactonase | 65.436 | -4.992 |
| PP_3379 | *kguE* | epimerase | 67.260 | -5.032 |
| PP_4844 | *urtD* | ABC transporter ATP-binding protein | 71.388 | -5.118 |
| PP_0273 | no_symbol | hypothetical protein | 109.363 | -5.733 |
