# Supplementary material for: Transcriptome remodeling of Pseudomonas putida KT2440 during mcl-PHAs synthesis: effect of different carbon sources and response to nitrogen stress
Source: J Ind Microbiol Biotechnol. 2018 May 7;45(6):433–46. doi: 10.1007/s10295-018-2042-4 (PMC6028892; doi:10.1007/s10295-018-2042-4)
Supplement: Supplementary file 2 — Supplementary material S2. Significantly differentially expressed genes of Pseudomonas putida KT2440 grown on oleic acid. (PDF 112 kb) [file 10295_2018_2042_MOESM2_ESM.pdf]

Journal of Industrial Microbiology & Biotechnology

Transcriptome remodeling of *Pseudomonas putida* KT2440 during mcl-PHAs synthesis: effect of different carbon sources and response to nitrogen stress

Justyna Mozejko-Ciesielska[1], Tomasz Pokoj[2], Slawomir Ciesielski[2]

Correspondence: Justyna Możejko-Ciesielska, Department of Microbiology, Faculty of Biology and Biotechnology, University of Warmia and Mazury in Olsztyn, Oczapowskiego 1A, 10-719 Olsztyn, e-mail: justyna.mozejko@uwm.edu.pl

Supplementary material S2. Significantly differentially expressed genes of *Pseudomonas putida* KT2440 grown on oleic acid.

Locus tag	Gene name	Description	Fold Change	log_2Fold Change
PP_1705	*nirB*	nitrite reductase large subunit	818.754	9.677
PP_2092	*nasA*	nitrate transporter	307.032	8.262
PP_1706	*nirD*	nitrite reductase	266.473	8.057
PP_4841	*urtA*	urea ABC transporter substrate-binding protein	93.311	6.543
PP_1703	no_symbol	assimilatory nitrate reductase/sulfite reductase	70.432	6.138
PP_4842	*urtB*	urea ABC transporter permease	67.328	6.073
PP_2090	*cobA*	uroporphyrinogen-III C-methyltransferase	55.862	5.803
PP_4843	*urtC*	urea ABC transporter permease	52.519	5.714
PP_4844	*urtD*	ABC transporter ATP-binding protein	40.896	5.353
PP_2093	no_symbol	two-component system response regulator NasT	36.519	5.190
PP_2688	no_symbol	hypothetical protein	34.960	5.127
PP_2686	no_symbol	transglutaminase domain-containing protein	31.253	4.965
PP_2687	no_symbol	hypothetical protein	26.633	4.735
PP_2091	no_symbol	serine/threonine-protein kinase	26.274	4.715
PP_4631	no_symbol	hypothetical protein	22.337	4.481
PP_5073	no_symbol	hypothetical protein	20.431	4.352
PP_2843	*ureA*	urease subunit gamma	19.285	4.269
PP_3213	no_symbol	ABC transporter substrate-binding protein	18.963	4.245
PP_1707	no_symbol	D-isomer specific 2-hydroxyacid	17.940	4.165

		dehydrogenase family protein		
PP_2844	*ureB*	urease subunit beta	13.384	3.742
PP_4738	no_symbol	hypothetical protein	11.725	3.551
PP_3214	no_symbol	hypothetical protein	11.651	3.542
PP_2708	no_symbol	hypothetical protein	11.483	3.521
PP_2710	no_symbol	hypothetical protein	11.285	3.496
PP_5593	no_symbol	hypothetical protein	10.934	3.450
PP_2846	*ureE*	urease accessory protein	9.2359	3.207
PP_0135	no_symbol	hypothetical protein	9.212	3.203
PP_5560	no_symbol	hypothetical protein	9.183	3.199
PP_2712	no_symbol	hypothetical protein	9.178	3.198
PP_1833	no_symbol	hypothetical protein	9.097	3.185
PP_2842	*ureD*	urease accessory protein	8.954	3.162
PP_2847	*ureJ*	urease accessory protein UreJ	8.035	3.00
PP_3208	no_symbol	Pdr/VanB family oxidoreductase	7.794	2.962
PP_3266	no_symbol	hypothetical protein	7.685	2.942
PP_3930	no_symbol	hypothetical protein	7.625	2.930
PP_5051	no_symbol	hypothetical protein	7.563	2.919
PP_3209	no_symbol	isoxanthopterin deaminase	7.357	2.879
PP_2848	*ureF*	urease accessory protein	7.285	2.864
PP_2404	no_symbol	hypothetical protein	7.232	2.854
PP_2676	no_symbol	substrate-binding protein	7.143	2.836
PP_4615	no_symbol	membrane protein	6.800	2.765
PP_1503	no_symbol	hypothetical protein	6.475	2.695
PP_3743	no_symbol	hypothetical protein	6.280	2.650
PP_0797	no_symbol	hypothetical protein	6.106	2.610
PP_2685	no_symbol	hypothetical protein	5.943	2.571
PP_2845	*ureC*	urease subunit alpha	5.912	2.563
PP_2677	no_symbol	hypothetical protein	5.655	2.499
PP_2709	no_symbol	long-chain fatty acid--CoA ligase	5.486	2.455
PP_4531	no_symbol	hypothetical protein	5.354	2.420
PP_2711	no_symbol	short-chain dehydrogenase/reductase family oxidoreductase	5.118	2.355

PP_3524	no_symbol	hypothetical protein	4.841	2.275
PP_2675	no_symbol	cytochrome c-type protein	4.806	2.264
PP_4855	no_symbol	osmotically-inducible lipoprotein OsmE	4.735	2.243
PP_5628	no_symbol	hypothetical protein	3.894	-2.300
PP_3788	no_symbol	non-ribosomal peptide synthetase	6.146	-2.847
PP_1788	no_symbol	hypothetical protein	6.398	-2.893
PP_3785	no_symbol	hypothetical protein	7.427	-3.108
PP_3781	no_symbol	oxygen-independent coproporphyrinogen III oxidase family protein	7.713	-3.163
PP_3784	no_symbol	hypothetical protein	8.564	-3.314
PP_3782	no_symbol	hypothetical protein	14.268	-4.050
PP_3783	*syrB*	syringomycin biosynthesis protein 2	15.352	-4.156
